# Under-representation of WHO Africa region in HBV clinical trials: The field advances, but in which direction?

**DOI:** 10.1016/S2468-1253(23)00315-1

**Published:** 2024-02-15

**Authors:** Marion Delphin, Khadija Said Mohammed, Louise O Downs, Sheila F Lumley, Elizabeth Waddilove, Dorcas Okanda, Nadia Aliyan, Marije Van Schalkwyk, Motswedi Anderson, Ponsiano Ocama, Tongai Maponga, Judith Torimiro, Collins Iwuji, Thumbi Ndung’u, Philippa C Matthews, Jantjie Taljaard

**Affiliations:** 1 The Francis Crick Institute, 1 Midland Road, London, UK; 2 Division of Biosciences, Faculty of Life Sciences, University College London, London, UK; 3 Nuffield Department of Medicine, Medawar Building for Pathogen Research, University of Oxford, South Parks Road, Oxford, UK; 4 Department of Infectious Diseases and Microbiology, John Radcliffe Hospital, Headley Way, Oxford, UK; 5 KEMRI-Wellcome Trust Research Programme, Kilifi, Kenya; 6 Kilifi County Hospital, Kilifi, Kenya; 7 Division of Infectious Diseases, Department of Medicine, Stellenbosch University / Tygerberg Academic Hospital, Cape Town, South Africa; 8 Botswana Harvard AIDS Institute Partnership, Princess Marina Hospital, Gaborone, Botswana; 9 Africa Health Research Institute, Durban, South Africa; 10 Makerere University College of Health Sciences, Kampala, Uganda; 11 Division of Medical Virology, Stellenbosch University / National Health Laboratory Service Tygerberg Business Unit, Cape Town, South Africa; 12 Molecular Biology Laboratory, Chantal Biya International Reference Centre for AIDS Research (CIRCB), Yaounde, Cameroon; 13 Department of Global Health and Infection, Brighton and Sussex Medical School, University of Sussex, Brighton, UK; 14 Division of Infection and Immunity, University College London, London, London, UK; 15 Department of Infectious Diseases, University College London Hospital, London, London, UK

## Abstract

The WHO Africa region bears a disproportionate burden of morbidity and mortality related to chronic Hepatitis B Virus (HBV), accounting for an estimated 70% of new infections worldwide. We investigated the extent to which HBV clinical trials (CT) represent populations in this region. Through analysis of CT registries, we identified 1804 CT amongst which <1% were based in WHO Africa. There is no evidence that this is improving over time. The diversity of new interventions evaluated in WHO Africa is low, and industry sponsorship limited, with studies of HBV comparing poorly to other endemic infectious diseases (malaria, HIV and SARS-Cov-2). This field has been neglected, leading to profound health inequities for people living with HBV in WHO Africa. HBV CT are urgently needed to evaluate efficacy of newly discovered therapeutics and to ensure that interventions can be equitably distributed and deployed as they become available.

## Introduction

HBV infection is a major public health concern and combating it is now enshrined in the United Nations (UN) Sustainable Development Goals (1). Specific high-profile elimination targets set out by Global Health Sector Strategies and the World Health Organization (WHO) aim to reduce new hepatitis infection by 90% and deaths by 65% by 2030 (2). The WHO Africa region bears a disproportionate burden of Chronic Hepatitis B (CHB), with 75 million chronic carriers, 71000 deaths each year, and an estimated 70% of new HBV infections (1), due to a sustained lack of investment in prevention, diagnosis, treatment, infrastructure, policy, advocacy and education (3).

CHB damages the liver, resulting in inflammation, fibrosis, cirrhosis and/or hepatocellular carcinoma (HCC). HCC is now one of the leading causes of cancer deaths in the WHO African region, being the top cause of cancer mortality in men (4). Two main tools are currently approved to reduce the burden of HBV and associated liver disease, (i) a safe and efficient subunit vaccine, available in monovalent and multivalent formulations, to prevent infection, and (ii) Nucleos(t)ide Analogue (NA) agents to control viral replication, reducing long-term complications and limiting transmission. Current national and international clinical guidelines (5–9) recommend that NA agents are only offered to a minority of individuals living with CHB, aiming interventions at those with the highest risk of complications and transmission. However, there is an increasing shift to relaxed eligibility criteria, such that antiviral agents will become more widely prescribed in diverse clinical scenarios (10). As NA therapy controls but does not clear HBV infection, novel antivirals permitting functional cure may also be required to achieve elimination targets worldwide.

As people living with CHB mostly encounter the virus at birth or in the early years of life, one of the international elimination targets is for <0·01% prevalence in children aged under five (2). To reach these goals, timely birth dose (BD) vaccine and/or antenatal NA prophylaxis of mothers living with CHB are recommended and have been demonstrated to be cost effective compared to antiviral treatment of adults with CHB (11). Most countries are on track to meet these paediatric targets or have already achieved them (3). However, 26/32 countries projected not to meet these criteria are in WHO Africa, despite the region achieving a reduction in HBV seroprevalence from 7·8 to 2·7% in children in the last 30 years (1–3). Gavi has recently announced re-engagement in the HBV BD vaccine program (12), which was paused due to the COVID-19 pandemic; combined with lack of commitment and investment from regional/national stakeholders, the neglect of this intervention may have been more impactful on African lives than the pandemic itself (13).

It is of crucial importance that new interventions for HBV are evaluated worldwide, with particular emphasis on populations most at risk of infection and long-term liver disease. These outcomes are influenced by host genetics, HBV genotypes, co-infections, drug susceptibility and distinct transmission patterns which vary by geographic locations (14). Within the WHO-Africa region, specific consideration may be needed to investigate the impact of HBV transmission routes and age at acquisition of infection, population genetics, dietary aflatoxin exposure (15), HIV co-infection (16), and high prevalence of HBV genotypes A and E. In Africa, HCC typically arises a decade earlier than in Western countries, and patients present late with advanced malignancy, with a median survival at diagnosis of only 2·5 months (vs 24 months in the United States) (15). In addition, the aspartate aminotransferase (AST) to platelet ratio index (APRI) used to assess liver fibrosis under-estimates cirrhosis in African populations (17), and HBV genotype E has been characterised by lower viral loads whilst being equally - or even more – oncogenic compared to other genotypes, which could lead patients to fall below the cut-off threshold for treatment eligibility despite being at risk (18).

In addition to these biological and virological influences, evidence is needed to explore the accessibility, acceptability, and affordability of interventions by setting, accounting for local policy, culture, resources, and infrastructure. All these factors need to be considered to evaluate and implement treatment interventions that are efficient and can be equitably implemented.

We therefore set out to determine the extent to which HBV CT represent populations in the WHO Africa region.

## Methods

### Search strategy and selection criteria

**Figure 1:**
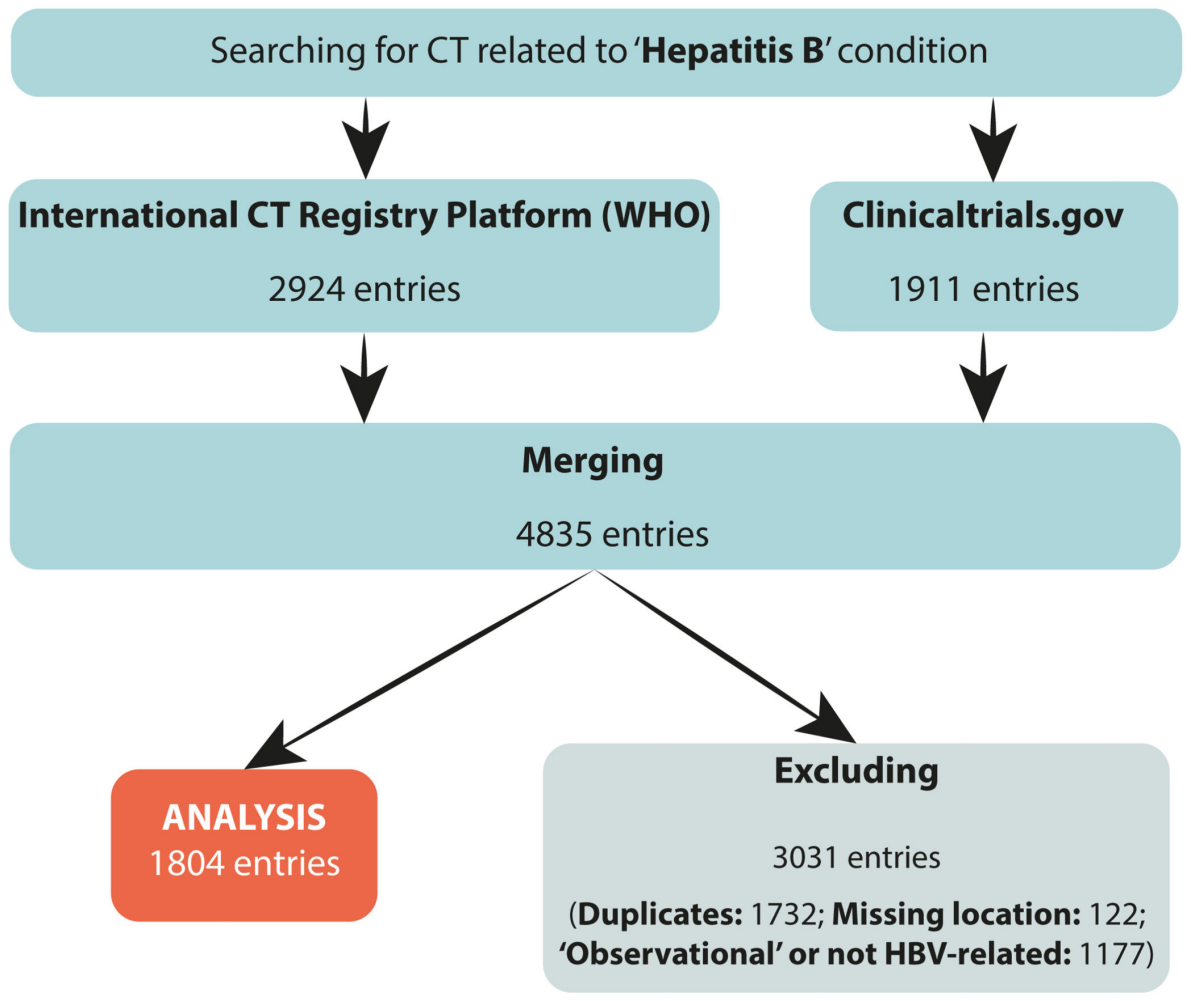
Protocol and decision tree associated with investigation of HBV Clinical Trials (CT). The International CT Registry Platform (ICTRP) from the World Health Organization (WHO), and ‘ClinicalTrials.gov’ registries were searched. Search criteria were ‘Hepatitis B’. Data from each registry were merged, and CT were excluded to remove (1) duplicates, (2) studies with missing location data or (3) those that did not meet criteria for HBV-related interventional CT, (i.e: studies categorized as ‘observational’ or patients were not infected with HBV or were not part of the evaluation of an antiviral drug against HBV). The material is presented according to the standardized PRISMA reporting criteria.

We searched the International CT Registry Platform (ICTRP) from the WHO, and ‘ClinicalTrials.gov’ registries for CTs reported in English, without limitation by start year, on May 29, 2023. For our primary search, we used ‘Hepatitis B’ as the only search term (Figure 1). Similarly, we undertook secondary searches for ‘HIV’, ‘malaria’ or ‘SARS-CoV-2’ to act as comparators (Appendix page 2). We obtained CTs starting as early as 1983, and from 2020 for SARS-CoV-2.

For each pathogen, data from these registries were merged and duplicates removed, based on Trial ID. CT were only eligible for inclusion when focused on the pathogen of interest, but we included studies both of individuals with the relevant infection and of healthy volunteers (e.g., for HBV studies, we permitted ‘CHB’, ‘HBV’, ‘Hepatitis B’ and ‘healthy’, with the latter reflecting enrolment for phase I CT for evaluation of HBV-specific interventions). Studies categorized as ‘observational’ (i.e., no intervention) or with missing location data were excluded. Conflicts over inclusion were discussed between at least two authors. For each registry and pathogen, we extracted the following CT variables: study ID, study title, condition of the participants, interventions, sponsors/collaborators, phase(s) of the trial, start year, location and URL. Details of authors who undertook the primary search and data extraction are listed in ‘Authors’ contributions’ section. We collected data in Microsoft Excel (version 16.73) and populated our record based on the relevant fields in the online databases. The spreadsheet was locked prior to final analysis and made available to the public (19).

### Data analysis and presentation

We sorted CTs by WHO region, based on their registered location(s) (Variable ‘Location’ from the registries), using R (Version 4.2.1; script made publicly available (20)). For studies that were conducted in more than one WHO region, we counted the study once in our overall dataset, but logged each region separately (hence the number of studies reported by region adds up to more than the total number of studies). Seroprevalence numbers used in this analysis were from the Global Burden of Disease report published in 2019 (1). We investigated changes in distribution of CT over time, using the ‘Start Year’ variable, and types of CT using the ‘Interventions’ variable.

To gather data on industry involvement, we searched the ‘Sponsor/Collaborators’ variable for companies involved in HBV drug development, based on the Hepatitis B Foundation drug watch list (21). We identified the top ten industry partners from trials databases and collected data for the following companies: GSK, Gilead Science, Roche, Bristol Myers, Novartis Pharmaceuticals, Sanofi, Assembly Biosciences, Janssen, Arbutus and Bukwang Pharmaceuticals.

Figures were generated using GraphPad Prism (v. 9.5.1) or Adobe Illustrator (v. 27.5).

## Results

We identified 2924 and 1911 HBV CT through the ICTRP and ClinicalTrials.gov registries respectively. After removal of duplicates, 3103 unique studies were included, of which 1804 met the eligibility criteria (Figure 1). Using similar search and inclusion/exclusion strategies, these databases recorded 1521, 7496 and 11886 unique studies of malaria, HIV and SARS-CoV-2 respectively (Appendix page 2).

### The distribution of HBV clinical trials is poorly matched to regions with the highest prevalence of infection, with particular neglect of the WHO Africa region

**Figure 2:**
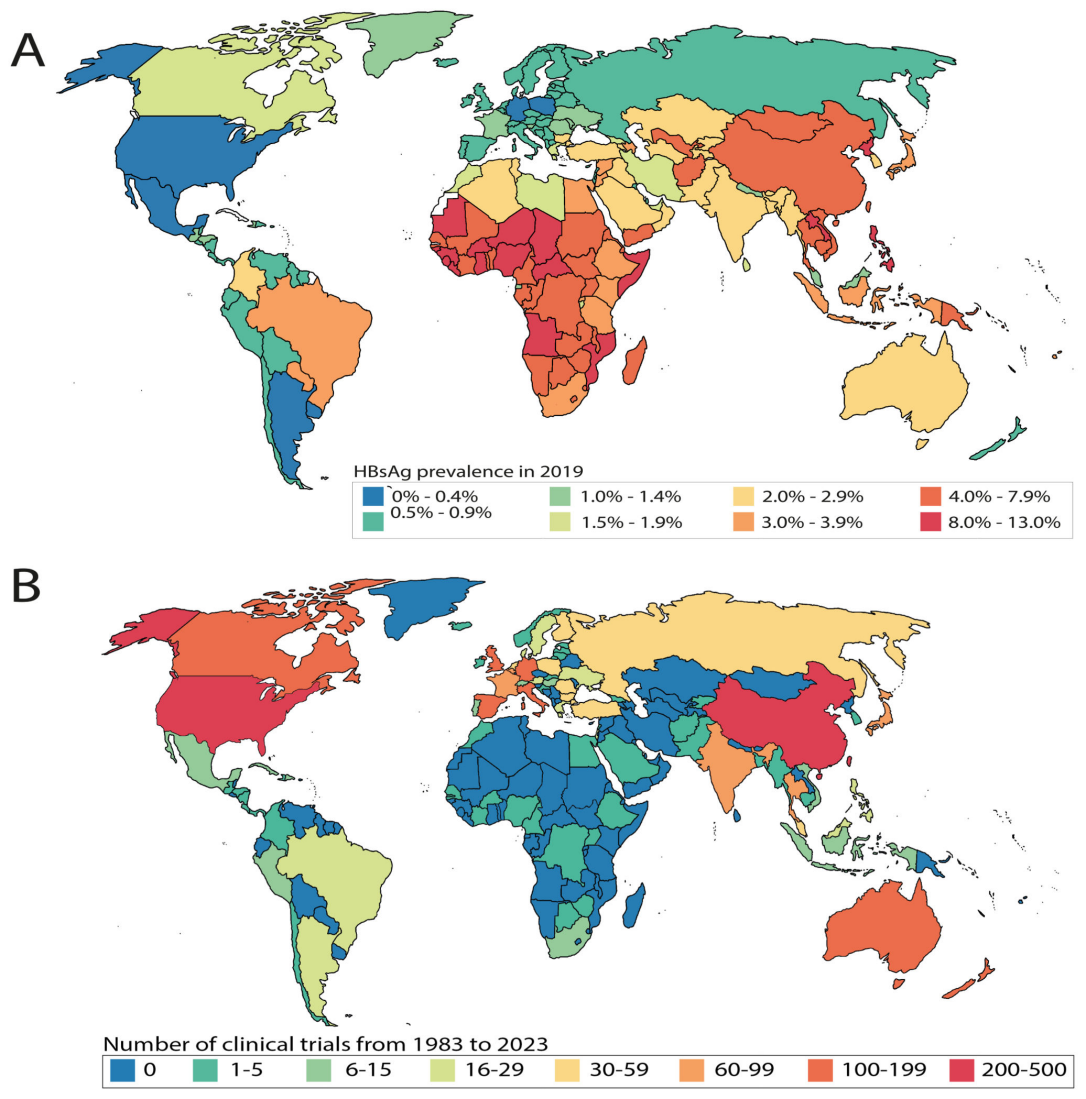
Contrasting geographical distribution of HBV seroprevalence and HBV clinical trials (CT). **(A)** Map representing geographical distribution of HBsAg seroprevalence in 2019, figure adapted from (1). **(B)** Map representing numbers of CT associated with HBV in each country from 1983 to March 2023.

After sorting by geographical location and WHO region, we compared the global distribution of HBV CT with regional Hepatitis B surface Antigen (HBsAg) prevalence. Seroprevalence of HBsAg is the highest in WHO Western Pacific region (7·1%), followed by the Africa region (6·5%), with lower prevalence in other regions (Eastern Mediterranean (3·1%), South-East Asia (3·1%), Americas (1·2%) and Europe (1·1%)) (Figure 2A) (1).

Appropriately reflecting the high seroprevalence of HBV infection in the region, the WHO Western Pacific had the highest number of CT (accounting for 1025/1804 trials; 56·8% of the total) (Figure 2B). Conversely, representation of HBV CT in the WHO Africa region fails to account for the high prevalence of infection, as 25·9% of global HBV infection is in this region (1,22), but only 0·99% (18/1804) of registered worldwide CT record involvement of populations in WHO Africa (Figure 2B). Europe (539/1804; 29·9%) and Americas (321/1804; 17·8%) were the second and third most represented regions in terms of HBV CTs, although the overall population HBsAg seroprevalence is only ~1% (1). South-East Asia and the Eastern Mediterranean, that have HBsAg seroprevalence of around 3·1%, represent 10·0% (181/1804) and 3·21% (58/1804) of global CT, respectively.

### Phase III and IV CT are lacking in the WHO Africa region

**Figure 3:**
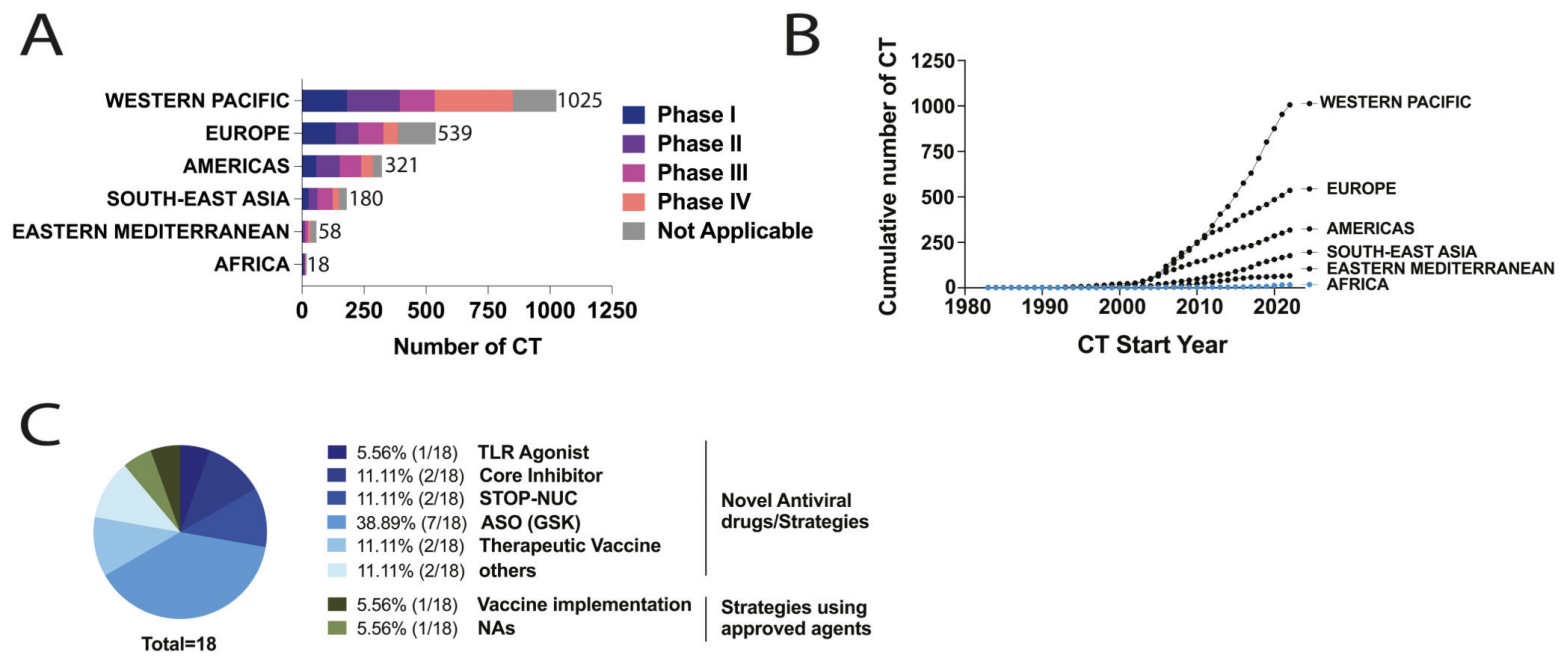
Hepatitis B Clinical Trials (CT) by location, over time, and according to agent(s) under review, between 1983 and 2023. **(A)** CT phases according to WHO geographical region. Total numbers are presented for each bar. ‘Not Applicable’ refers to CT where the information was not disclosed in the databases. **(B)** Cumulative number of CT per year by region. **(C)** Pie chart to show agents represented in 18 CT in the WHO Africa region. NA - nucleos/tide analogue; TLR - Toll-Like Receptor; STOP-NUC - STOP NUCleotides; ASO - Anti-Sense Oligonucleotides; GSK - GlaxoSmithKline Pharmaceutical Ltd.

We next investigated the phases of CT being undertaken in different regions. In WHO Africa, amongst 18 registered CT, six CT were in phase I and II, representing 1·64% and 1·74% of worldwide CT for these phases respectively, with only three CTs in phases III/IV (Figure 3A; Appendix page 2). Most phase IV studies were in the Western Pacific (73.4%, 315/429), while earlier phases were more equally distributed between other regions (Appendix page 2). As later study phases are associated with implementation and long-term follow-up of sustainability, efficiency and side-effects of interventions, upscaling these studies in the WHO Africa region is crucial.

### Studies in WHO Africa have not increased over time, despite the approval of new agents for HBV infection

Reviewing CT spanning >40 years (1983 to 2023), we investigated any changes in distribution of studies over time (Figure 3B). Whilst the number of CTs for most regions increased shortly after the year 2000, aligning with FDA approval of the first oral antiviral agent for HBV (lamivudine), this was not the case for the WHO Africa region. A further step up in the number of trials in the Western Pacific region occurred at the time of FDA approval of tenofovir for HBV in 2009. These data suggest that the neglect of investment in HBV CTs in the WHO Africa region has been consistent through time and is not improving despite a range of new potential agents and new strategies for implementation of existing drugs (21).

### Studies in WHO Africa region fail to represent a diversity of new drug agents in trials for HBV infection

Among the 18 CT we identified in WHO Africa region, seven (38·9%) were associated with the trial of one GSK Anti-Sense Oligonucleotide (ASO) compound (23) (Figure 3C). Other CT were testing Core Allosteric Modulators (2/18, 10·5%), therapeutic vaccines (2/18, 10·5%) (24), ‘STOP-NUC-trial’ strategies (2/18, 10·5%), Toll-Like Receptor (TLR) agonists (1/18, 5·3%), NA studies (1/18, 5·3%), and vaccine implementation (1/18, 5·3%). Lastly, two studies were associated with other antiviral agents but did not lead to any published results or were never completed. Altogether, only eight compounds out of the 76 mentioned on the Hepatitis B Foundation drug watch (21) were trialled in the global region with the second highest HBsAg seroprevalence.

### Neglect of HBV CT in the WHO Africa region is disproportionate compared to studies tackling other endemic infectious diseases

In order to understand if the lack of CT in WHO Africa region is associated with a regional lack of research capacity associated with personnel, expertise, resources, and infrastructure, we reviewed equivalent data for malaria (where 235·65M/247M cases occur in WHO African countries, 95% of worldwide cases) (25) and HIV (for which 25·7M/37·9M global cases are in WHO Africa, 67·8%) (26). SARS-CoV-2 infections accounted for a lower disease burden in WHO Africa region than in other regions (8.986M/767,364M, 1·17%) (27), but COVID-19 trial data can also provide insights into trial capacity and international collaboration.

**Figure 4:**
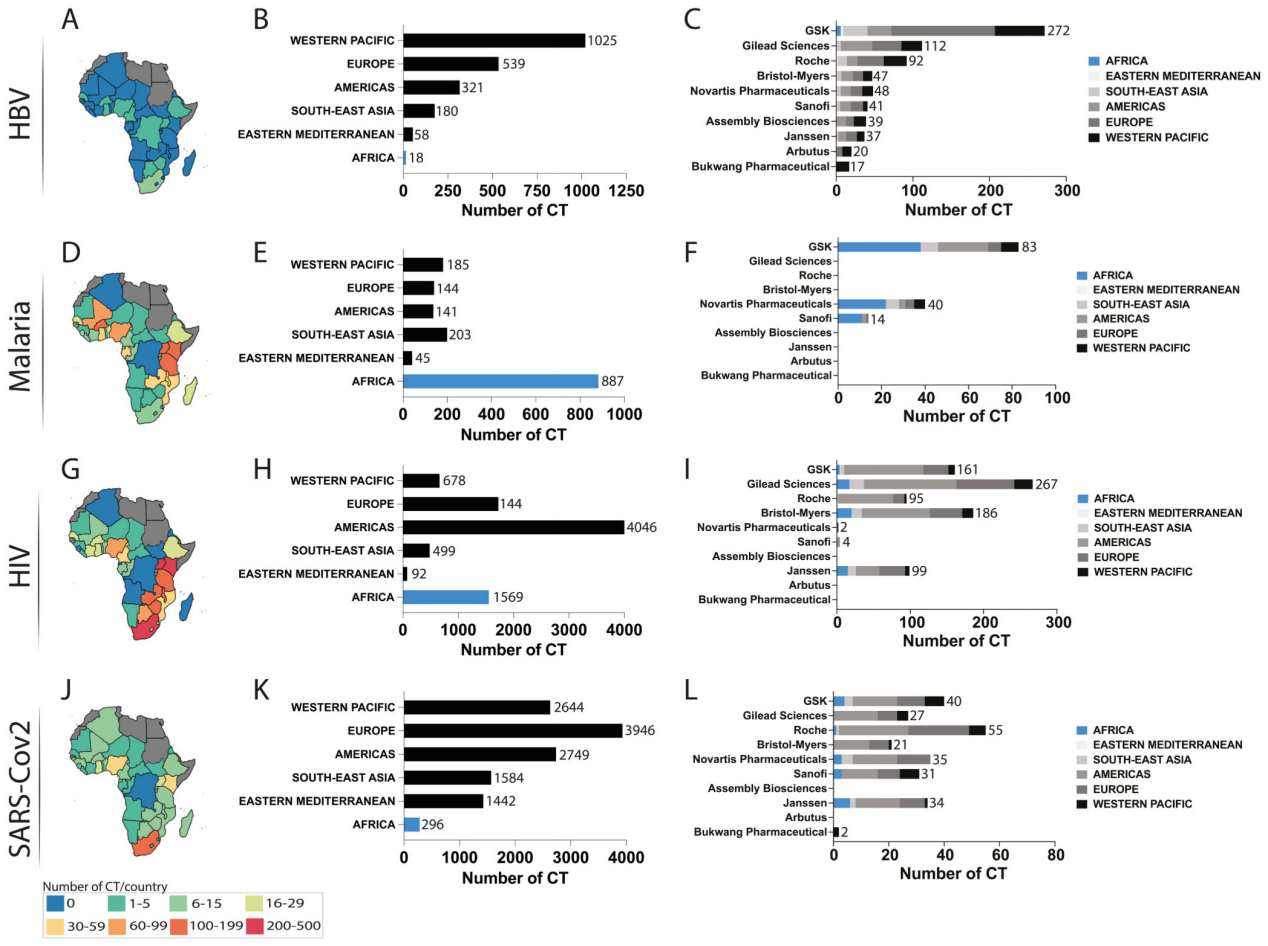
Comparison of Clinical Trial (CT) distribution in the WHO Africa region (between 1970 and 2023) for four pathogens HBV (panels A-C), malaria (panels D-F), HIV (panfels G-I) and SARS-CoV-2 (panels J-L). First column shows maps of WHO Africa region (countries shown in colour) representing number of CT per country (panels A, D, G, J); second column shows number of CT identified per WHO region (panels B, E, H, K); third column shows main sponsors of HBV CT by WHO region (panels C, F, I, L). In bar plots, total numbers are displayed for each bar.

Across these four infectious agents, CT for HBV in the WHO Africa region contributed the lowest proportion of all studies at 0·99% (18/1804), compared to 20·9% for HIV, 2·5% for SARS-Cov2 and 58·0% for malaria (Figure 4.B, E, H, K). This demonstrates African capacity for CT, highlighting potential to deliver leadership for other infectious diseases that are of particular relevance to regional populations, building on relevant expertise and infrastructure that are already in place, as well as aiming to scale up capacity over time.

### Industry involvement in HBV CT in WHO African region is inadequate

Among the top ten industries in the HBV field working on antiviral therapeutics, only GSK had a documented association with HBV-related CT in WHO Africa region (Figure 4C). However, pharma sponsors are clearly present in the region, as evidenced through CT involvement in studies of malaria, HIV and SARS-CoV-2, for which we identified involvement of GSK, Novartis, Sanofi, Gilead, Bristol-Myers, Janssen and Roche (Figure 4. F, I, L; Appendix page 3).

**Figure 5:**
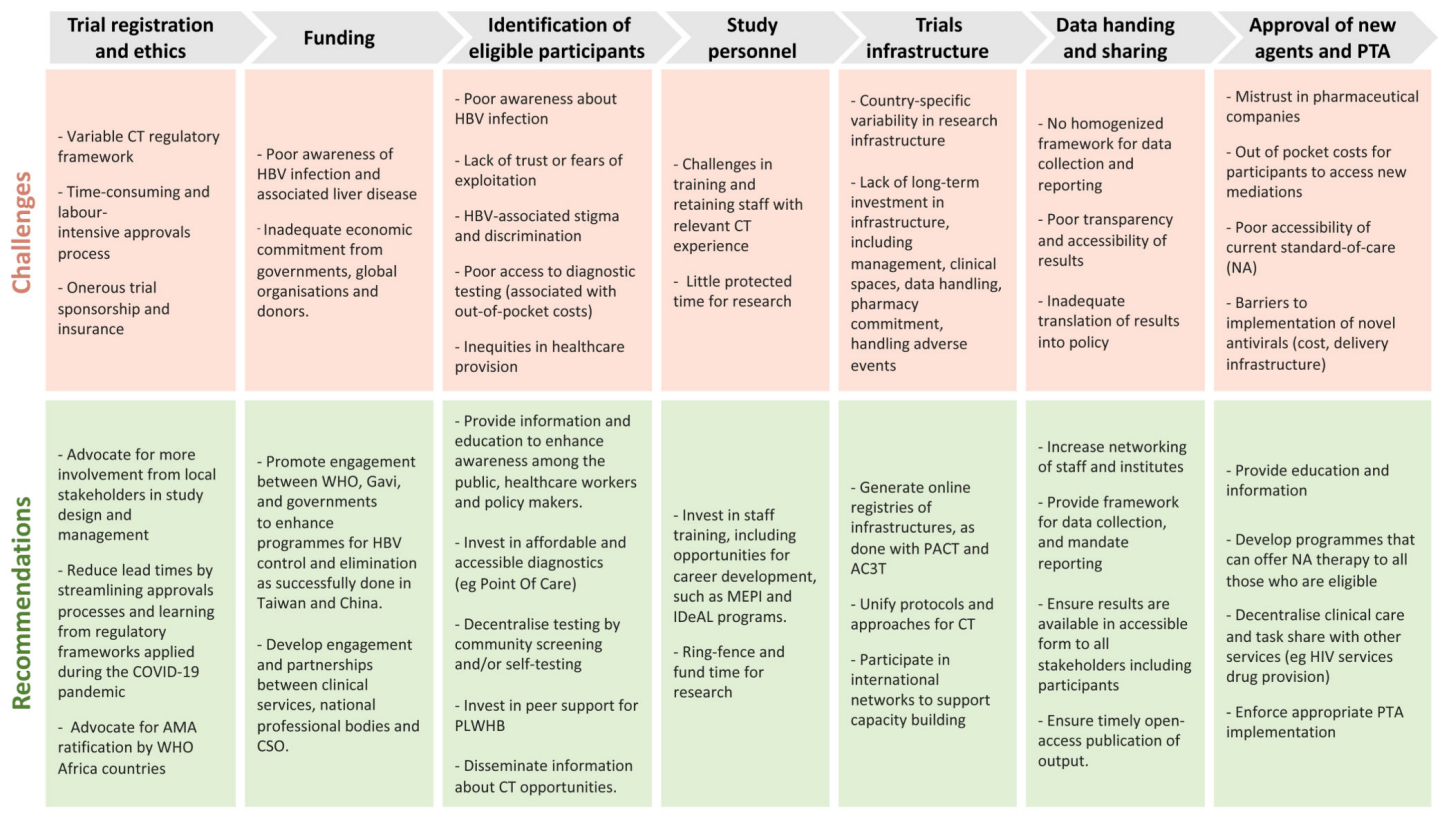
Challenges and suggested recommendations associated with establishing Clinical Trials (CT) for HBV infection in the WHO Africa region. AC3T: African Consortium for Cancer Clinical trials (29); AMA: African Medicine Agency (30); CSO: Civil Society Organisation; IDeAL: Initiative to Develop African research Leaders (31); MEPI: Medical Education Partnership Initiative (32); PACT: Pan African CT registry (33); PLWHB: People Living With Hepatitis B, PTA: Post-Trial Access.

Following our investigation, we have summarized the challenges and recommendations for future CT in figure 5 (28).

## Discussion

### Summary of findings

HBV is a neglected pathogen, only recognised as a global pandemic by WHO in 2011, with people living with HBV calling themselves ‘the forgotten people’ (34). Although there has been a scale-up of translational HBV research in some areas of the world, especially evident in the Western Pacific, populations in the WHO-Africa region have been consistently under-represented. This suggests that the location of trials is often driven by resources rather than clinical need, exacerbating substantial health inequities and reducing the chances of success in meeting HBV global elimination targets, as people most in need of new interventions are least likely to be able to access them.

The distribution of different trial phases may provide insights about resources and infrastructure; phase I and II trials in WHO Africa region demonstrate the presence of scientific skills and expertise, while the costs of phase III/IV may explain the relative paucity of these larger clinical studies (35). However, the burden of infection and associated morbidity, alongside specific risk factors (host and viral genetics, diet, co-infections), should make African populations a priority for phase III and IV studies.

### Opportunities for translational HBV research in WHO Africa

Lack of recruitment accounts for 25-30% failure of CT worldwide (36), leading to significant losses for investors. Enrolling participants for CT is thus strategic not only from biological and social perspectives, but also from an economic point of view in delivering robust, adequately powered studies in a timely way that can feed directly back into enhancing the health of local populations.

In recent years, a number of bodies have been established to harmonise, strengthen and develop CT activity in WHO Africa (extensively discussed in other papers (30,35)). As a result, during the COVID-19 pandemic many countries in the region were able to demonstrate an agile response with re-deployment of existing platforms alongside rapid scale-up of infrastructure and investment (37,38). For example, the Sisonke program in South Africa brought together government, national stakeholders, foundations and industry during the deadliest wave of COVID-19, to trial and provide vaccines to healthcare workers (48). As well as delivering crucial protection to the population, this activity delivered the first and largest study evaluating Ad26.COV2.S vaccine in a real-world population, with results that informed global strategy and implementation (39). This clearly demonstrates the presence of clinical research capacity. However, many countries in WHO Africa do not have the infrastructure present in South Africa, and unfortunately this flexible and responsive use of resources has not yet been similarly applied to tackle the similarly devastating impact of HBV infection.

### Next steps in scaling up HBV CT in the WHO Africa region

Enhanced CT activity in WHO Africa will come hand-in-hand with decolonisation of global research, such that leadership and governance are owned by the country where the work is being done (Figure 5). Trials should be driven by local priorities, replacing those with little population health impact (such as allergic rhinitis or overactive bladder) with studies that tackle key local and regional challenges including malaria, tuberculosis (TB) and neglected tropical diseases (40). Development of HBV CT activity needs to be undertaken in partnership with local stakeholders, clinical teams, community leaders, and people with lived experience of HBV infection (41) (Figure 5). There are diverse barriers and challenges for provision of holistic HBV care; those designing and leading trials must be sensitive to barriers that may include poor awareness and education, lack of trust and fear of exploitation, stigma, and out-of-pocket costs (42,43) (Figure 5).

Implementation studies are also crucial to develop an evidence base for scaling up HBV diagnosis and treatment, for example exploring the potential gains that can be made through ‘triple elimination’ programmes which tackle perinatal HBV alongside HIV and syphilis or linking to the provision of HIV Pre-Exposure Prophylaxis (PrEP). Such infrastructure already provides NA therapy, and links to laboratory capacity, which could bring a wider and more cost-effective approach to the clinical care of HBV (44). Clinical trials can be associated with risks (45) and it is therefore imperative that early phase trials in particular are conducted in healthcare environments that are capable of investigating the causality for potential drug toxicity and mitigating the risks. Allocating resources for scale up of clinical and research infrastructure, alongside robust ethics and governance oversight, is essential, bringing wide benefits that are applicable to all CT.

Predicting accessibility, acceptability, and affordability of the intervention trialled is also crucial to ensure a quick and efficient rollout. For HBV this is a particular challenge, as most people living with HBV are undiagnosed (associated with late presentations of liver disease including advanced malignancy), attributed to neglect of advocacy/education, but also stigma and discrimination, and poor access to diagnostic tools. Decentralisation of testing by community screening (46) and/or self-testing (47) has already been proven efficient and is thus recommended (Figure 5). For the few aware of their status, the limited access to investigation with laboratory tests and imaging makes follow-up and disease stratification nearly impossible. However, access to antivirals has been widened in the WHO Africa region, especially in countries with concomitant HIV epidemic/programs (through access to better laboratory facilities and NA drugs). There are also logistical and practical barriers to vaccine uptake, including particularly poor access to monovalent vaccination (as BD doses and to protect adults in high-risk groups), as well as challenges related to vaccine hesitancy which may be associated with social, political, religious, and cultural factors (48).

Since 2000, Post-Trial Access (PTA) stipulations are included in the Declaration of Helsinki, with an amendment in 2013 to request governments to take responsibility for research participants and their communities. However, specific guidelines are unclear about PTA implementation, and PTA is not often delivered in practice, leaving participants with a feeling of abandonment and lack of understanding, such that they may revert to traditional practice rather than adopting new interventions (49). Enhanced CT activity must thus come hand in hand with better long-term implementation to safeguard ethical research, retain public trust and reach HBV elimination by providing education, follow-up, and access to interventions including new treatment.

### Mandates for Funding

We here present evidence of neglect of the WHO Africa region by industries developing HBV antivirals. In addition, the field is suffering a broader neglect, as non-profit organisations have also not stepped up to invest. Thus, it is now clear that financial support and greater commitment from governments, international institutions, civic society, and donors is required to achieve elimination, saving many lives whilst having direct and indirect cost effects yielding large economic benefits, as discussed recently (50). Some of these strategies were already applied in Taiwan (51) and China (52), leading to succesful decrease of HBV seroprevalence and liver cancer incidence (Figure 5). There is currently a lack of commitment by governments to commission supplies of the current (low-cost) NA antivirals for HBV which are required as a foundation for trials of new agents (many of which will only be offered to patients who are already virologically suppressed on standard-of-care therapy). It is reasonable to expect that governments of countries hosting trials should commit to the procurement of NA therapy as an essential clinical intervention as well as to provide a platform for new therapy; in due course similar agreements will be needed to support access to new medications for HBV (i.e in line with PTA), albeit at generic pricing levels.

### Challenges and limitations

Our data represent a broad overview of CT activity, and do not provide higher resolution insights into the specific populations represented by clinical research. There are many differences in populations, infrastructure, and resources between settings in the WHO Africa region and regulatory frameworks differ by location; accordingly, the barriers to establishing successful CT are diverse, and not captured through this review. Equitable representation is difficult even in regions where HBV research is already established, as CT are unlikely to reflect the most vulnerable population groups, such as asylum seekers, in whom HBV is disproportionately enriched (e.g., estimated seroprevalence 10% as a result of populations being displaced from areas of high/intermediate HBV endemicity, compared to general population (typically ~1%)) (34). The ‘inclusion health’ agenda highlights a pressing need for the development of research and clinical service provision that better reflects the needs of these vulnerable and under-served communities (34).

Some of the limitations of our approach are related to the registries. As there are slight differences in the information recorded by the two registries, we focused only on data that are common to both. Entries into these databases are dependent on the provision of source data, and many values are missing (especially CT locations) or erroneous (e.g., entries in the wrong field). Data are also entered in diverse ways, with heterogeneity potentially making analysis difficult. Details of investment from non-industry sources (not-for-profit organisations, charities, academic institutes, government funds, etc) are not available in either registry, and would be informative to obtain a higher resolution picture on HBV funding.

CTs are difficult to implement in settings where there is poor deployment of approved, existing standard-of-care interventions, including education, vaccination, access to diagnostic testing, laboratory assays and imaging, and treatment for those who are eligible. Enhancing the scope, reach and representation of CT is dependent on the urgent scale-up of investment in accessible and equitable clinical infrastructure.

## Conclusions

There is a clear neglect of investment in HBV-focused CT activity in the WHO Africa region. Diverse trials, ranging from exploration of new drugs through to implementation studies, are urgently needed to reduce the morbidity and mortality of chronic HBV infection. Support is required from governments, industry, and donors, alongside a scale up of community advocacy representing people with lived experience of HBV. CT in the WHO Africa region will provide an evidence foundation to support the optimum use of existing interventions, to evaluate the safety and efficacy of newly discovered anti-HBV compounds, and to ensure that these can be equitably distributed and deployed as they become available.

## Supplementary Material

Appendix
