# Supplementary material for: Under-representation of WHO Africa region in HBV clinical trials: The field advances, but in which direction?
Source: Lancet Gastroenterol Hepatol. Author manuscript; Available in PMC 2024 May 29. (PMC7616036; doi:10.1016/S2468-1253(23)00315-1)
Supplement: Appendix [file EMS196196-supplement-Appendix.pdf]

THE LANCET
Gastroenterology & Hepatology

Supplementary appendix

This appendix formed part of the original submission and has been peer reviewed. We post it as supplied by the authors.

Supplement to: Delphin M, Mohammed KS, Downs LO, et al. Under-representation of the WHO African region in clinical trials of interventions against hepatitis B virus infection. *Lancet Gastroenterol Hepatol* 2024; published online Feb 14. https://doi.org/10.1016/S2468-1253(23)00315-1.

Supplementary material associated with the article 'Under-representation of WHO Africa region in HBV clinical trials: The field advances, but in which direction?'

Table of content:

Supplementary Figure 1……………………………..………………………….….....2
Supplementary Table 1……………………………………………………………......2
Supplementary Table 2………………………………….……………………….....3

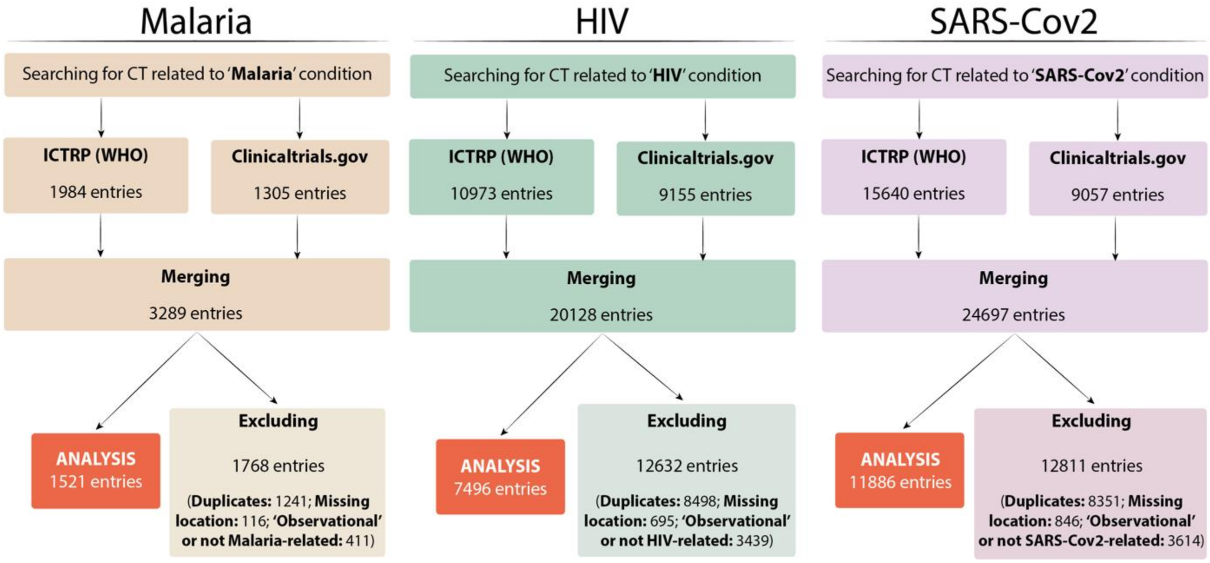

Supplementary Figure 1: Protocol and decision tree associated with investigation of Malaria, HIV and SARS-Cov2 CT. The ICTRP and 'clinicaltrial.gov' registries were searched. Data from each registry were merged, and CT were excluded when (1) we identified and removed duplicates, (2) there was missing location data or (3) they were not meeting the criteria for specific pathogen-related interventional CT, i.e: CT were categorized as 'observational' or patients were not infected with the pathogen of interest or were not part of the evaluation of an antiviral drug against the pathogen.

	**Phase I**	**Phase II**	**Phase III**	**Phase IV**	**N.A**
**Total**	301	345	310	429	419
**Africa**	5 (1·66%)	6 (1·74%)	1 (0·32%)	2 (0·47%)	4 (0·95%)
**Americas**	57 (18·9%)	95 (27·5%)	86 (27·7%)	45 (10·5%)	37 (8·83%)
**Eastern Mediterranean**	4 (1·33%)	9 (2·6%)	12 (3·87%)	8 (1·86%)	25 (5·97%)
**Europe**	137 (45·5%)	91 (26·4%)	100 (32·3%)	59 (13·7%)	152 (36·3%)
**South-East Asia**	26 (8·64%)	36 (10·4%)	61 (19·7%)	26 (6·06%)	32 (7·64%)
**Western Pacific**	178 (59·1%)	213 (61·7%)	141 (45·5%)	315 (73·4%)	181 (43·2%)

Supplementary Table 1: Table of different phases of HBV CT per WHO region. For each region, numbers of CT and the percentage they represent over the total HBV CT for this phase (row 1 'total') are displayed. 'N.A': refers to CT where the database logged the trial phase as 'Not Applicable', or this field was 'Not Available' (blank).

The numbers in each column do not add up to the total number of trials reported in the first row because some trials include data for more than one region. Percentages are calculated based on each region as a proportion of all trials in the given phase, and again because each trial may represent >1 region the percentage for each column adds up to >100%.

		1	2	3	4	5	6	7	8	9	10
**Africa**	HBV	7	0	0	0	0	0	0	0	0	0
	HIV	4	17	0	20	0	0	0	15	0	0
	Malaria	38	0	0	0	22	11	0	0	0	0
	SARS-Cov2	4	0	1	0	3	3	0	6	0	0
**Americas**	HBV	31	41	14	15	13	14	13	10	1	0
	HIV	108	126	75	93	1	3	0	32	0	0
	Malaria	23	0	0	0	3	2	0	0	0	0
	SARS-Cov2	16	16	25	13	16	13	0	16	0	0
**Europe**	HBV	135	38	34	13	15	16	10	14	7	0
	HIV	34	79	15	44	0	1	0	35	0	0
	Malaria	6	0	0	0	4	1	0	0	0	0
	SARS-Cov2	10	7	22	7	12	8	0	9	0	0
**Eastern Mediterranean**	HBV	3	0	2	1	0	0	0	0	0	0
	HIV	1	0	0	0	0	0	0	0	0	0
	Malaria	0	0	0	0	0	0	0	0	0	0
	SARS-Cov2	0	0	0	0	0	0	0	0	0	0
**South-East Asia**	HBV	32	6	12	6	6	5	0	3	0	0
	HIV	5	20	2	14	0	0	0	11	0	0
	Malaria	8	0	0	0	6	0	0	0	0	0
	SARS-Cov2	3	0	1	0	4	0	0	2	0	0
**Western Pacific**	HBV	65	27	30	12	14	6	16	10	12	17
	HIV	9	25	3	15	1	0	0	6	0	0
	Malaria	8	0	0	0	5	0	0	0	0	0
	SARS-Cov2	7	4	6	1	0	7	0	1	0	2

Supplementary Table 2: Table of different funders/sponsors of HBV, Malaria, HIV and SARS-CoV-2 Clinical Trial (CT) per WHO region. Columns headings correspond to the following: 1: GlaxoSmithKline, 2: Gilead Science, 3: Roche, 4: Bristol Myers, 5: Novartis, 6: Sanofi, 7: Assembly Biosciences, 8: Janssen, 9: Arbutus and 10: Bukwang Pharmaceutical.
